# Recent Dermatological Treatments for Stevens-Johnson Syndrome and Toxic Epidermal Necrolysis in Japan

**DOI:** 10.3389/fmed.2021.636924

**Published:** 2021-07-29

**Authors:** Ming-Hsiu Hsieh, Tomoya Watanabe, Michiko Aihara

**Affiliations:** ^1^Department of Dermatology, Yokosuka Kyosai Hospital, Yokosuka, Japan; ^2^Department of Environmental Immuno-Dermatology, Yokohama City University Graduate School of Medicine, Yokohama, Japan

**Keywords:** intravenous immunoglobuline, methylprednisolone pulse therapy, Stevens-Johnson syndrome, toxic epidermal necrolysis, treatment

## Abstract

Stevens-Johnson syndrome (SJS) and toxic epidermal necrolysis (TEN) are serious conditions characterized by necrosis of the skin and mucus membranes, and are mainly caused by medication and infections. Although the exact pathomechanism of SJS/TEN remains unclear, keratinocyte death is thought to be triggered by immune reactions to these antigens. While there is no established therapy for SJS/TEN, corticosteroids and intravenous immunoglobulin (IVIG) have been utilized as immunomodulator. We previously conducted a study to evaluate the efficacy of IVIG therapy in Japanese patients with SJS/TEN. IVIG was administered at a dosage of 400 mg/kg/day for 5 consecutive days as an additional therapy with systemic steroids. Prompt amelioration was observed in seven of the eight patients. All patients survived without sequelae. Recently, we retrospectively analyzed 132 cases of SJS/TEN treated in our two hospitals. The mortality rates in the patients treated with methylprednisolone pulse were 0% (0/31) for SJS and 7.0% (3/43) for TEN, and 0% (0/10) in the TEN patients treated with methylprednisolone pulse in combination with IVIG. These results suggest that early treatment with high-dose steroids, including methylprednisolone pulse therapy, and IVIG together with corticosteroids are possible therapeutic options to improve the prognosis of SJS/TEN.

## Introduction

Stevens-Johnson syndrome (SJS) and toxic epidermal necrolysis (TEN) are rare but serious conditions characterized by necrosis of the skin and mucus membranes, and are most frequently caused by medication, and less frequently by infections, mainly *Mycoplasma pneumoniae*. The symptoms of SJS and TEN are high fever, necrosis of the skin and mucous membranes resulting in erythema, blisters/erosions and enanthema including severe eye lesions, and oral and genital bleeding erosions (1). Because TEN mostly occurs in patients with SJS, followed by rapid progression, these two disorders have been defined as the same disease spectrum and are classified on the basis of the percentage of body surface area (BSA) affected: <10% of BSA in SJS, between 10 and 30% of BSA in SJS/TEN overlap, and >30% of BSA in TEN (2). In addition to mucocutaneous lesions, SJS/TEN affects various organs and can induce life-threatening complications such as multiorgan failure. Severe mucosal involvement of the ocular epithelium and respiratory tract is accompanied by serious sequelae. The global mortality rates for SJS and TEN are as high as 5–10 and 20–40%, respectively (3–6).

The exact pathomechanism of SJS/TEN remains unclear. Keratinocyte death is thought to be caused by cytotoxic T cells and natural killer (NK) cells, and triggered by soluble Fas ligand (FasL), perforin/granzyme B, and granulysin produced by such activated cells (7–10). TNF-α, a major proinflammatory cytokine is highly expressed in the skin of SJS/TEN and suggested to be responsible for extensive skin necrosis (11). It was also demonstrated that the necroptosis pathway contributes to keratinocyte death in SJS/TEN through the interaction between monocyte-derived annexin A1 and formyl peptide receptor 1 expressed on keratinocytes (12). A number of pharmacogenetic studies of human leukocyte antigen (HLA)-associated drug hypersensitivity have recently been performed. In SJS/TEN induced by some drugs, such as anticonvulsants and allopurinol, T cell activation may be caused by the binding of specific drug antigens and HLA molecules with higher binding affinities to the antigens (11).

Treatment for SJS/TEN is the prompt cessation of the causative agent and supportive therapy. Many studies have described the efficacy of immunomodulator for SJS/TEN. Among them, systemic corticosteroids and intravenous immunoglobulin (IVIG) have been utilized in clinical practice (10, 13, 14). Previous reports discuss the drawback of corticosteroid therapies from the point of increased infection such as sepsis and no benefit at mortality. However, a recent systematic review and meta-analysis of systemic immunomodulating therapies for SJS/TEN concluded that the treatment of systemic corticosteroids significantly improved the prognosis of TEN compared to supportive care (13). In addition, Liu et al. reported a significantly lower mortality rate in the steroid treatment group than the mortality rate predicted by the SCORTEN (14). Treatment with IVIG increased after Viard et al. reported favorable outcomes with IVIG (10). In addition, a combination of corticosteroids and IVIG was reported as a more promising strategy than corticosteroids and IVIG alone (15). In the ophthalmologic field, it has been reported that treatment with steroid pulse therapy (500 mg to 1 g/day for 3 days) and topical corticosteroid in the early stage of onset is effective for acute severe ocular involvement with corneal ulceration associated with TEN (16). Furthermore, Kim et al. demonstrated that early administration of IVIG or high dose systemic corticosteroids could improve acute ocular involvement (17). However, the effects remain controversial.

Recently, cyclosporine administration was reported as a new therapeutic approach. Some studies have shown that cyclosporine reduces mortality in patients with TEN in combination with systemic corticosteroids in SJS/TEN patients (13). In another study, a comparison of the observed vs. expected mortality risk ratio of 49 cyclosporine-treated patients was performed (18) using the severity-of-illness scoring system for TEN (SCORTEN), which is widely used as a standard prognostic tool (19). The analysis showed a reduction in mortality risk with cyclosporine administration. On the other hand, the results of an epidemiological study including 174 patients did not show any beneficial effect of cyclosporine (3 mg/kg/day) in patients with SJS/TEN (20). Therefore, further studies are needed to evaluate the efficacy of cyclosporine for SJS/TEN treatment.

More recently, a randomized controlled trial was conducted to compare the effects of TNF-α antagonist (etanercept) vs. traditional corticosteroids in 96 SJS/TEN patients. The results showed that both etanercept and corticosteroids decreased the SCORTEN-based predicted mortality rate, but etanercept further reduced the skin-healing time in moderate-to-severe SJS/TEN patients compared with corticosteroids (21). This result shows the possibility that anti-TNF-α biological agents are an alternative for the treatment of SJS/TEN. There are presently insufficient data to discuss the efficacy of TNF-α antagonists.

Herein, we review the efficacy of treatments for SJS/TEN, focusing on IVIG and high-dose corticosteroids, and outline our previous study of IVIG treatment additionally administered with corticosteroids, and our recent retrospective analysis of the efficacy of methylprednisolone pulse therapy for SJS/TEN. Finally, we discuss treatments for SJS/TEN recently introduced in Japan.

## IVIG Therapy for SJS/TEN

### IVIG Therapy in SJS/TEN Treatment in the World

In 1998, Viard et al. found that IVIG preparations containing Fas-blocking antibodies inhibit Fas-FasL interaction from keratinocyte apoptosis by blocking Fas receptors, and the capacity abrogated after the depletion of antibodies (10). They also reported an open, uncontrolled pilot study of 10 SJS and TEN patients receiving IVIG (0.2~0.75 g/kg per day for 4 consecutive days). Disease progression was interrupted within 24 to 48 h, and rapid skin healing with favorable outcomes was also noted, proving their *in vitro* study (10). Because IVIG provides inhibition of Fas-FasL-mediated keratinocyte apoptosis and other multiple immune modulators that are still under investigation, the application of IVIG in the treatment of SJS/TEN increased over time and gradually become the main approach, even though the effects are still controversial. We summarized previous reports on the efficacy of IVIG (Table 1). A prospective non-comparative evaluation was performed from 1999 to 2000 in 34 patients (26% SJS, 15% SJS/TEN overlap, 59% TEN) under a total dose of 2 g/kg of IVIG within 2 days. The predicted death from SCORTEN was 8.2 deaths, and the actual mortality was 11 deaths without significant difference from prediction. They also pointed out that the progression of skin lesions was not arrested after IVIG administration (22). These negative results suggest that the destruction of epidermal cells is provoked by several apoptotic pathways and the blockage of Fas-FasL interaction is insufficient.

**Table 1:** IVIG and systemic corticosteroids therapy for SJS/TEN in the world.

**References**	**Study design**	**Sample size and classification, study period**	**Treatment**	**Author conclusion**
IVIG therapy for SJS/TEN				
Viard et al. (10)	Prospective, open, uncontrolled, multicenter	1 SJS, 4 SJS/TEN overlap, 5 TEN, N/A	IVIG (0.2~0.75 g/kg per day for 4 consecutive days)	Effective, response within 24–48 h
Bachot et al. (22)	Prospective non-comparative SCORTEN based comparison	9 SJS, 5 SJS/TEN overlap, 20 TEN, 1999~2000	IVIG (total dose of 2 g/kg within 2 days)	Ineffective
Tran and Sidhu (23)	Retrospective chart review	18 SJS, 6 SJS/TEN overlap, 18 TEN, 2000~2009, 2010~2017	(1) Skin and supportive, (2) Oral corticosteroids (3) IVIG alone (4) Oral corticosteroids and IVIG IVIG doses (1–3 g/kg/day)	Effective, improvement in mortality in IVIG groups
Lee et al. (24)	Retrospective, a single referral center	28 SJS/TEN overlap, 36 TEN1. 2003-12, 2010	IVIG dosage (1) <3 g/kg (2) larger than 3 g/kg	Ineffective in mortality between dosage
Antoon et al. (25)	Retrospective Cohort Study, multicenter data from the Pediatric Health Information System	774 SJS, 124 TEN(IVIG only: 56 TEN)(IVIG and steroid: 15 TEN)(Steroid only: 12 TEN)2008~2015	167 steroids only, 229 IVIG only, 153 both IVIG and steroids	Ineffective, bias in distribution of severity
Yang et al. (26)	Retrospective, SCORTEN based comparison	(1) 10 SJS, 35 TEN, 1993~2001 (2) 8 SJS, 12 TEN, 2001~2007	(1) 1–1.5 mg/kg/day methylprednisolone (2) 2 g/kg of IVIG (0.4 g/kg/day for 5 days) with a combination of corticosteroids	Effective in mortality, disease progression and time of hospitalization in combined group
Chan and Cook (27)	Retrospective, a single referral center	10 SJS, 6 SJS/TEN overlap, 26 TEN, 2006~2016,	(1) Skin and supportive 3 SJS, 1 SJS/TEN, 2 TEN (2) Corticosteroids alone 2 SJS, 0 SJS/TEN, 5 TEN (3) IVIG alone 1 SJS, 4 SJS/TEN, 11 TEN (4) corticosteroids and IVIG 4 SJS, 1 SJS/TEN, 8 TEN	Effective in mortality in combined group
Micheletti et al. (15)	Retrospective, multicenter of 18 academic medical centers	110 SJS, 158 SJS/TEN overlap, 79 TEN, 2000~2015	(1) Skin and supportive, (2) Corticosteroids, mean (148 mg prednisone for 9.8 days) (3) IVIG alone, mean (1 g/kg/day for 3 days) (4) Oral corticosteroids and IVIG (5) Cyclosporine or tumor necrosis factor inhibitor	Ineffective. However, concluded co-administration of corticosteroids and IVIG deserving further prospective trials
Yang et al. (28)	Retrospective, SCORTEN based comparison, a single referral center	141 SJS, 19 SJS/TEN overlap, 53 TEN, 2008~2018	Systemic corticosteroids and IVIG (mainly 0.4 g/kg/day for 5 days)	Effective in mortality without significance
Pham et al. (20)	Retrospective, a single institution	13 SJS/TEN, N/A	(1) Etanercept alone (2) IVIG for 3 days and Etanercept (3) Without Etanercept (Etanercept 50 mg)	Effective under Etanercept treatment without statistical significance
**An open-label, multicenter, single-arm study of IVIG therapy in Japan**				
Aihara et al. (29)	Prospective, open-label, multicenter, single-arm study	5 SJS, 3 TEN, N/A	Systemic corticosteroids and IVIG (400 mg/kg/day for 5 consecutive days, total 2 g/kg)	Effective without mortality in all patients
**Systemic corticosteroid therapy for SJS/TEN**				
Yamane et al. (5)	Retrospective, two university hospitals	52 SJS, 35 TEN, 2000~2013	Steroid pulse therapy in combination with plasmapheresis and/or IVIG	Effective, lower than SCORTEN based mortality
Liu et al. (14)	Retrospective, SCORTEN based comparison, a single referral center	18 SJS, 23 SJS/TEN overlap, 29 TEN, 2008~2015	(1) Low-dose group (≤ 2 mg/kg/d) (2) High-dose group >2 mg/kg/d (5 mg prednisone or 4 mg methylprednisolone or 5 mg hydroprednisone or 0.75 mg dexamethasone).	Supporting the use of systemic corticosteroids for SJS/TEN.
Araki et al. (16)	Prospective, observational case series	4 SJS, 1 TEN, N/A	Steroid pulse therapy; 500 or 1000 mg/day for 3 to 4 days. Additional steroid (prednisolone 40~60 mg/day)	Effective, early steroid pulse therapy improving ocular symptoms
Hirahara et al. (30)	Retrospective	3 SJS, 2 SJS/TEN overlap, 3 TEN, 2008~2015	Methylprednisolone pulse therapy (1,000 mg/d for 3 consecutive days), Oral prednisolone at 0.8–1 mg/kg/d	Effective, reduction in the mean levels of IFN-γ, TNF-α, and IL-6
Watanabe et al. (31)	Retrospective	75 SJS, 53 TEN, 2000~2019	Methylprednisolone pulse therapy (500–1,000 mg/day of methylprednisolone for 3 days), prednisolone equivalent 1 mg/kg/day, part of patients combined with IVIG and/or plasmapheresis	Effective, the mortality rate are lower than the global mortality rates
Sunaga et al. (32)	Retrospective, a nationwide survey in Japan,160 institutions	315 SJS, 174 TEN, 2016~2018	(1) 37.8 % high-dose steroid alone (2) 29.2% pulse therapy followed by tapering (3) 11.7% high-dose steroid plus IVIG (4) 13.7% steroid pulse therapy plus IVIG (5) High-dose steroid (0.80–1.21 mg/kg) (6) IVIG (0.36–0.43 g/kg for 5 days)	Effective in mortality in high-dose steroid followed by pulse group

*IVIG, intravenous immunoglobulin; N /A, no data; SJS, Stevens–Johnson syndrome; TEN, toxic epidermal necrolysis; SJS /TEN, SJS-TEN overlap. The classification was made according to the original study. If there was no classification in the study, we classified according to the classification of Roujeau and Stern, 1999 from the raw data as SJS:detachment of TBSA <10%, 10% < SJS /TEN <30%, and <30%: TEN.*

*Review articles were not listed in this table*.

Although the efficacy of IVIG remains uncertain, several case series or reviews suggested that high-dose IVIG (more than 2 g/kg total dose) is associated with a tendency for better improvement. A recent retrospective review in Australia comparing data between 2000–2009 (mainly corticosteroid) and 2010–2017 (mainly IVIG) of 42 SJS/TEN patients revealed improvement in mortality in IVIG groups (50 vs. 27.3%) (23). However, not every study shows positive results for high-dose IVIG. A retrospective review divided 64 patients (44% with SJS/TEN overlap, 56% with TEN) into IVIG dosage <3 g/kg and larger than 3 g/kg groups from 2003 to 2010, and the mortality was 31% without significant differences in mortality between high and low dosage groups (24).

Since better progression and less mortality tendency were reported after the administration of IVIG in some studies, physicians tend to give severe patients IVIG under the expectation of good progression, and thus may indirectly cause bias in the results of retrospective studies. A 7-year retrospective review of children with SJS/TEN evaluated 898 pediatric patients from the Pediatric Health Information System between 2008 and 2015. They reported longer length of stay in the IVIG alone and IVIG combined corticosteroid groups, and an increasing odds risk of mechanical ventilation use in the IVIG alone group. In that article, they also pointed out the deviation of distribution of severity in that more patients were diagnosed with TEN in the IVIG and combined groups (25).

For the suppression of multiple apoptotic pathways that cause keratinocyte death, the combination of IVIG with other immunomodulators is an emerging issue for researchers. A 14-year retrospective review from China evaluated 45 patients (35 with TEN, 10 with SJS) treated with 1–1.5 mg/kg/day methylprednisolone between 1993 and 2001, and 20 patients (12 with TEN, 8 with SJS) receiving a total dose of 2 g/kg of IVIG (0.4 g/kg/day for 5 days) with a combination of corticosteroids from 2001 to 2007. The combination group of patients with TEN presented a tendency for decreased mortality rate in comparison with the corticosteroid alone group. The time to interrupt the disease progression and time of hospitalization were reduced significantly in the IVIG combined with corticosteroid group, but the time of tapering off steroids did not differ between the SJS and TEN groups (26). A 10-year retrospective review from Australia evaluated 42 patients with SJS/TEN from 2006 to 2016, and divided the patients into IVIG alone, IVIG combined with corticosteroid, corticosteroid alone, and supportive care groups. Although the dosage of IVIG and corticosteroid and the start of treatment deviated, there was no death in the group of IVIG combined with corticosteroid group with statistically significance. There was no statistically significant difference between different doses of IVIG in survival outcome (27). Favorable results without significance were also revealed in recent studies. In the United States, a multicenter study of 18 academic medical centers reviewed 377 adult patients with SJS/TEN between 2000 and 2015. Although there was no significant difference in mortality between all the subgroups of treatment, the authors concluded that co-administration of corticosteroids and IVIG (10.7% mortality) deserves further prospective trials (15). A similar conclusion was also mentioned in a retrospective review between 2008 and 2018 in a Chinese hospital, which included corticosteroid with IVIG as the first-line treatment. A total of 213 SJS/TEN patients receiving systemic corticosteroids and IVIG (mainly 0.4 g/kg/day for 5 days, for progressive condition) had lower mortality (3.8%) than the prediction of SCORTEN (8.6%) without significance (28). The latest network meta-analysis of review articles in the Journal of the American Academy of Dermatology evaluated 66 studies, involving 2,079 SJS/TEN overlap and TEN patients from 1999 to 2019. Although it was reported that none of the included systemic immunomodulating therapies reduced mortality rates in patients with SJS/TEN overlap and TEN, based on standardized mortality ratio, the combination of corticosteroids and IVIG significantly reduced the standardized mortality ratio and hinted at the synergistic effects of targeting different pathways (33).

In addition to corticosteroids, accompanied by the use of the TNF-α antagonist, IVIG combined with etanercept (50 mg) was evaluated at a single institution in California from 2005 to 2018. Although there was no difference in mortality (15.4 vs. 10%), and lower ICU stay (6.9 vs. 15.1 days) and lower total LOS (9.8 vs. 16.4 days) were without statistical significance, compared with their previous cohort review of IVIG alone, the SCORTEN, affected total body surface area and disease presentation were worse than those in the IVIG alone group. This indicates that there is also little improvement in the treatment effect of a combination of IVIG and other immunomodulators (34).

The history of the application of IVIG in SJS and TEN spans only 20 years, and most of the literature is limited to case series or retrospective reviews because of the rarity of SJS/TEN. IVIG still has its position in the treatment of SJS/TEN and further prospective evaluation of its application deserves to perform in combination with other immunomodulators.

### An Open-Label, Multicenter, Single-Arm Study of IVIG Therapy in Japan

To evaluate the efficacy of IVIG therapy, we previously conducted an open-label, multicenter, single-arm study in Japanese adult patients with SJS/TEN (29). Enrolled patients showed progressive or unchanged symptoms with systemic steroids and judged not to respond sufficiently to systemic steroids. IVIG therapy was administered at a dosage of 400 mg/kg/day for 5 consecutive days (total 2 g/kg) as an additional therapy to systemic steroids in patients (five patients with SJS and three patients with TEN). All patients were observed on day 20 of IVIG treatment. The primary efficacy end-point was the response on day 7 of IVIG treatment, and ophthalmic lesions, lip/oral lesions, cutaneous lesions, and general condition were evaluated using our rating scale system. As a result, all of the patients survived and the efficacy on day 7 of the IVIG was 87.5% (seven out of eight patients). Prompt amelioration was observed in skin lesions and mucocutaneous lesions in patients in whom IVIG therapy was effective. Ophthalmic lesions were observed in seven patients at baseline and improved in six patients without sequelae, but little in one non-responder with TEN on day 7. No serious side effects were reported in any patient. In the seven responders, IVIG was started from 3 days to 13 days after the onset of cutaneous symptoms, while 23 days after the onset in the non-responders treated with steroids during the time. This time difference suggests that it is important to perform additional IVIG therapy promptly when a patient does not respond well to corticosteroid therapy. Analysis of the clinical course showed the possibility that early treatment with IVIG (400 mg/kg/day) administered for 5 consecutive days together with corticosteroids is effective in refractory SJS/TEN patients. Since only eight SJS/TEN patients participated, we could not show a significant difference between the predictive number of deaths (one patient) and our study (all survived).

In conclusion, we suggest that IVIG together with corticosteroids should be considered as a treatment modality for SJS/TEN patients, although a larger trial is needed to define the therapeutic efficacy of IVIG. After this study was performed in 2014, IVIG therapy was covered by health insurance for refractory SJS/TEN with steroid use in Japan.

## High-Dose Systemic Corticosteroid Therapy for SJS/TEN

### Review of Systemic Corticosteroid Therapy for SJS/TEN

Steroids bind to glucocorticoid receptors in the cytoplasm and act on the promoter regions of target genes to promote transcription of the related genes. In addition, steroids bind to transcription factors such as AP-1 and NF-κB and suppress the transcription of inflammatory cytokines (35). Because the glucocorticoid receptor in the cytoplasm is saturated in a dose-dependent manner and is 100% saturated at doses of prednisolone (PSL) over 100 mg/day, further escalation of PSL doses does not change the effect in the genomic mechanism (36). On the other hand, in the non-genomic mechanism, steroids bind to glucocorticoid receptors in the membrane and inhibit the arachidonic acid cascade via signaling, resulting in the suppression of enzyme activity and inflammatory responses. This function is fast-acting and has a steroid dose-dependent effect, such as in steroid pulse therapy and high-dose systemic corticosteroid therapy (36).

To evaluate the efficacy of systemic corticosteroid therapy, we summarized previous reports (Table 1). In the treatment of SJS/TEN, multiple previous studies reported that there was no difference in prognosis between supportive care and corticosteroid therapy because of the infection by immunosuppression of steroids (4, 37). On the other hand, in a systematic review and meta-analysis of 96 studies performed between 1990 and 2012, it was reported that three studies suggested the benefit of steroid treatment in prognosis, of which, one study significantly improved the prognosis of TEN compared with supportive care (13). Recently, a significantly lower mortality rate in the steroid treatment group than the mortality rate predicted by the SCORTEN has been reported (14). Furthermore, there are multiple previous studies that showed the possible usefulness of combination therapy with corticosteroids and IVIG as mentioned above (15, 26–28, 33, 38), even though steroid use alone did not show sufficient effects in those studies. Therefore, the therapeutic effect of steroids remains controversial.

In Japan, systemic steroid treatment has been mainly used for the treatment of SJS and TEN. Despite not being used worldwide, the use of methylprednisolone pulse therapy is increasing for severe and rapidly progressive cases based on the accumulation of individual case reports and epidemiological studies in Japan (5, 30). Skin lesions and enanthema usually start to recover within 3 days after initiating steroid pulse when the therapy is effective. Notably, it has been reported that steroid pulse therapy with concomitant use of topical betamethasone at disease onset greatly prevented ocular complications in Japanese patients with SJS/TEN (16). In the future, a randomized clinical trial of steroid pulse therapy compared with other treatments for SJS and TEN is required, although the rarity of these diseases precludes large-scale studies.

### Current Retrospective Analysis of Efficacy of Methylprednisolone Pulse Therapy for SJS/TEN

To evaluate the efficacy of steroid pulse therapy, we retrospectively analyzed 132 cases of SJS and TEN treated at Yokohama City University Hospital and Yokohama City University Medical Center between January 2000 and March 2019 (31).

A total of 128 patients with SJS and TEN including overlap (75 patients, 96.2% for SJS; 53 patients, 98.1% for TEN) were treated with corticosteroids. The mean age was 52.2 years for SJS (median, 54.0 years) and 57.3 years for TEN (median, 61.5 years). Of these, methylprednisolone pulse therapy (500–1,000 mg/day of methylprednisolone for 3 days) was performed in 31 (41.3%) patients with SJS and 43 (78.0%) for TEN to prevent the rapid progression of severe skin and mucocutaneous lesions. Other patients were treated with prednisolone or betamethasone (mostly prednisolone equivalent 1 mg/kg/day). In addition to systemic corticosteroids, patients with rapidly expanding skin detachment were treated with IVIG and/or plasmapheresis therapy. Plasmapheresis, mostly plasma exchange, was performed to remove the causative drugs and metabolites, and proinflammatory cytokines. Two patients with SJS were treated with IVIG in combination with steroid pulse therapy, while 13 patients with TEN were treated with IVIG and systemic corticosteroids, including 10 with steroid pulse therapy. Of these TEN patients treated with pulse and IVIG, the four most serious patients were added plasmapheresis therapy before IVIG, and as a result, all four patients recovered. Finally, the mortality rates for SJS and TEN were 1.3% (1/78) and 12.5% (6/54), respectively. These values are lower than the global mortality rates for SJS and TEN (3–6).

We further focused on the efficacy of methylprednisolone pulse therapy on mortality. The mortality rates in the patients treated with steroid pulse therapy with or without IVIG and/or plasmapheresis were 0% (0/31) for SJS and 7.0% (3/43) for TEN. In addition, the mortality rate was 0% (0/10) in patients with TEN treated with steroid pulse therapy in combination with IVIG or IVIG and plasmapheresis. Next, we evaluated the difference between the expected and actual numbers of deaths in patients with TEN using SCORTEN. There was no statistically significant difference in the SCORTEN score between the methylprednisolone pulse therapy group (2.14 ± 1.04) and other groups (except methylprednisolone pulse therapy) (2.67 ± 1.49). However, the actual number of dead cases in the steroid pulse therapy group (three cases) was lower than the predicted dead cases (9.3 cases), while the actual and predicted dead cases were almost equivalent in the other group (3 cases vs. 2.6 cases). As a result, the actual mortality rate (7.0%) was lower than the predicted mortality rate (21.6%) in the pulse group, although the difference was not statistically significant (Chi-squared test, *P* = 0.102) (Table 2A). Furthermore, in the methylprednisolone-treated cases, differences between the numbers of predicted and actual dead cases were observed in both the pulse group (3 cases vs. 5.8 cases) and pulse in combination with IVIG group (0 cases vs. 1.6 cases) (Table 2B) without statistical significance. Of note, sequelae of eye lesions were not observed in any surviving patients. In two TEN patients who died of sepsis, steroid pulse therapy started more than 7 days after the onset (13 and 14 days, respectively). This delay in the initiation of treatment might attenuate the effect of steroid pulse therapy and affect the prognosis. These results suggest that early steroid pulse therapy may be effective and contribute to the improved prognosis of SJS and TEN. The limitations of this study include various combinations of treatment with steroid pulse and a small number of cases treated with steroid pulse combined with IVIG.

**Table 2A:** Comparison of actual and predicted mortality in the treatment of methylprednisolone pulse.

**SCORTEN**	**Predicted mortality, *%***	**Patients, *n***	**Predicted death**	**Actual death**
0–1	3.2%	12	0.38	0
2	12.1%	14	1.69	0
3	35.3%	13	4.59	1
4	58.3%	3	1.75	2
≥ 5	90.0%	1	0.90	0
**Total**		43	9.32	3
**SMR**			0.32	

*The comparison of actual and predicted mortality rates associated with the treatment of TEN showed the predicted mortality to be 9.32 cases (21.7%) in all patients and the actual mortality to be three cases (6.98%). In this study, there were no significant differences between predicted and actual mortality (p = 0.1017, X^2^ test with Yates' continuity correction). SMR, standardized mortality ratio*.

**Table 2B:** Comparison of actual and predicted mortality between methylprednisolone pulse only and methylprednisolone pulse+IVIG.

**SCORTEN**	**Predicted mortality, *%***	**Methylprednisolone pulse only**			**Methylprednisolone pulse+IVIG**		
		**Patients, *n***	**Predicted death**	**Actual death**	**Patients, *n***	**Predicted death**	**Actual death**
0–1	3.2%	10	0.32	0	1	0.03	0
2	12.1%	9	1.09	0	7	0.85	0
3	35.3%	10	3.53	1	2	0.71	0
4	58.3%	0	0.00	2	0	0.00	0
≥5	90.0%	1	0.90	0	0	0.00	0
**Total**		30	5.84	3	10	1.59	0

*The comparison of actual and predicted mortality rates associated with methylprednisolone pulse with/without IVIG. Methylprednisolone pulse only group showed the predicted mortality to be 5.84 cases (17.7%) and the actual mortality to be 3 cases (9.1%). Methylprednisolone pulse+IVIG group showed the predicted mortality to be 1.59 cases (15.9%) and the actual mortality to be 0 cases (0%). IVIG, intravenous immunoglobulin*.

## Recent Treatments for SJS/TEN in Japan

Since randomized control studies are rarely performed due to the severity and rarity (3, 6) of STS/TEN, none of the abovementioned treatments have been established as the standard. Therefore, no evidence-based standardized guidelines for SJS/TEN treatment are currently available in the world. In Japan, guidelines for the management of SJS and TEN were established in 2009 and revised in 2016 by the Japanese Research Committee on Severe Cutaneous Adverse Reaction (JSCAR) supported by the Ministry of Health, Labor, and Welfare of Japan (39). In the guideline, high-dose systemic corticosteroids are recommended to start within 7 days after onset under appropriate infection control. Methylprednisolone pulse therapy is the first line of therapy, especially for highly progressing and more serious cases. Even though skin lesions are not widespread in SJS, high-dose systemic corticosteroids are recommended for patients with serious eye lesions to prevent ocular sequelae. After administration, steroids should be reduced gradually to prevent a return of the condition, especially eye lesions. On the other hand, continuation of the same amounts of corticosteroid without remarkable efficacy is not recommended because of uselessness and prevention of steroid side effects. Moreover, combination therapy with corticosteroids and IVIG is suggested as an additional therapy in patients with more serious conditions, progressing even with steroid therapy. Cyclosporine and TNF-α antagonists are not yet positively recommended in the guidelines because of insufficient empirical data.

The nationwide epidemiological survey (2016–2018) in Japan revealed that mortality rate was 4.1% for SJS and 29.9% for TEN, respectively (32). High-dose systemic corticosteroid therapy followed by steroid pulse therapy contributed to the greatest reduction in mortality when comparing the ratio of expected mortality to actual mortality (32). Furthermore, the incidence of ocular sequelae of SJS/TEN was 7.6% for SJS and 8.0% for TEN, which were less than previously reported (40).

## Conclusion and Perspective

In the current review, we describe the efficacy of IVIG treatment and IVIG in combination with corticosteroids as immunomodulating therapies for SJS/TEN. In addition, we demonstrated our recent epidemiological study of SJS/TEN patients treated in our hospitals during the past 19 years that showed the possible effects of methylprednisolone pulse therapy, and that in combination with IVIG in more serious cases it had an effect on mortality and sequelae, although the difference was not statistically significant. Therefore, we suppose that methylprednisolone pulse therapy and also in combination with IVIG therapy are possible modalities in patients with refractory and rapidly progressing SJS/TEN if they are performed at an early stage under appropriate infection control. A limitation of this study is that randomized control studies were not performed. In the future, administration of cyclosporine and a TNF-α antagonist with or without steroids may be considered as alternative treatment modalities for SJS/TEN. Further trials are required to define the therapeutic efficacies of these treatments in SJS/TEN.

## Author Contributions

MA contributed to determine the content of each section and edited the manuscript. All authors collected the data, wrote each section of the manuscript, and approved the submitted version.

## Conflict of Interest

The published IVIG study (28) was funded by Nihon Pharmaceutical, but none of the authors was financially supported by the company. The authors declare that the other SJS/TEN research was conducted in the absence of any commercial or financial relationships that could be construed as a potential conflict of interest.

## Publisher's Note

All claims expressed in this article are solely those of the authors and do not necessarily represent those of their affiliated organizations, or those of the publisher, the editors and the reviewers. Any product that may be evaluated in this article, or claim that may be made by its manufacturer, is not guaranteed or endorsed by the publisher.

## Abbreviations

HLA: Human leukocyte antigen
IVIG: Intravenous immunoglobulin
NK: Natural killer
SJS: Stevens-Johnson syndrome
TEN: toxic epidermal necrolysis: JSCAR, the Japanese Research Committee on Severe Cutaneous Adverse Reaction
SCORTEN: the severity-of-illness scoring system for TEN.
